# Disorder and compositional dependences in Urbach-Martienssen tails in amorphous (GeTe)_x_(Sb_2_Te_3_)_1−x_ alloys

**DOI:** 10.1038/s41598-019-42634-8

**Published:** 2019-04-15

**Authors:** K. V. Shportko

**Affiliations:** grid.466789.2 V.E. Lashkaryov Institute of Semiconductor Physics of NAS of Ukraine, Kyiv, Ukraine

## Abstract

This work focuses on the compositional dependences in parameters that govern the optical properties of (GeTe)_x_(Sb_2_Te_3_)_1−x_ amorphous alloys in the wide spectral range from above the phonons and to the inter-band electronic transitions. We studied the absorption edge fluctuations that are linked to the variations of the bandgap *E*_*g*_, the width of Urbach-Martienssen tails *E*_*U*_, the Tauc parameter *B*^*1/*2^, and average halfwidth <*FWHM*> of Raman bands in amorphous (GeTe)_x_(Sb_2_Te_3_)_1−x_ alloys at various temperatures. Obtained results reveal the compositional trends in the influence of the disordering on the absorption processes in studied alloys.

## Introduction

In recent years, pseudo-binary chalcogenide (GeTe)_x_(Sb_2_Te_3_)_1−x_ alloys (GSTs) have attracted much attention from the fundamental and applied points of view. GSTs relate to the class of phase-change materials and possess a rather unconventional set of properties. Optical and electrical properties inherent to amorphous and crystalline states of GSTs exhibit significant contrast^1–4^. In GTSs the optical dielectric constant is 70–200% larger for the crystalline than for the amorphous phases^3^. GSTs provide evidence for a disorder-driven metal–insulator transition^5^. Furthermore, crystallization in these materials can be extremely rapid and can occur in less than 100 ns^6^. These aspects may explain the interest to the properties of the crystalline phase of these compounds.

GST alloys are among the most promising materials for data storage applications. They are already successfully employed in rewriteable optical data storage (compact discs (CD), digital versatile discs (DVD) and Blue-ray Discs (BD))^7^. In addition, GSTs also have a unique potential as emerging non-volatile electronic memories. The phase change random access memory (PCRAM) is a possible successor for current memory devices such as dynamic random access memory (DRAM), static random access memory (SRAM) and flash memory^8^. Among its attractive features is the simple cell structure with high scalability; it is non-volatile, has a relatively high read/write operation speed (<10 ns), and a long cycle life (>10^12^ operations).

As GST alloys exhibit the contrast in optical properties between amorphous in crystalline states, they are also promising for non-volatile photonic applications, such as all-photonic memories, colour-rendering, and nano-pixel displays^9^. These applications employ the property contrast between amorphous and the crystalline states of GSTs, the long-term stability of the amorphous phase plays one of the crucial roles in reliability of corresponding devices.

In the recent work^10^, we studied the compositional dependences in the vibrational properties of amorphous GSTs using Raman spectroscopy at ambient temperature. It was found that the systematic compositional dependences in the intensities of characteristic Raman bands correlate with evolution of the concentration of different structural units present in (GeTe)_x_(Sb_2_Te_3_)_1−x_ alloys. To gain deeper insight into vibrational properties of amorphous and crystalline GSTs, the investigation of the temperature-dependent behaviour of phonon modes was performed^11^, which directly revealed a correlation between anharmonicity, vacancy concentration, and ordering.

While modeling the dielectric function of some phase-change alloys in the amorphous state^12^, it was shown that quite small deviations in *ε*_2_ can cause pronounced differences at the calculated reflectance spectra. Introduction an additional constant *ε*_2_ to the dielectric function could significantly improve the quality of the fit of the experimental data. It was assumed that additional constant *ε*_2_ corresponds to the contribution of absorption present in the amorphous samples studied. Analysis of the decrease of the additional constant *ε*_2_ upon cooling and annealing was interpreted as shrinking absorption by defect states, caused by a non-reversible decrease in the number of defect states upon relaxation. However this approach does not give one a chance to evaluate the width of the Urbach-Martienssen tails formed by localized and additional states. Furthermore, there is a lack of the information on the compositional dependences in the Urbach-Martienssen tails in amorphous phase-change alloys. The latter might bring insight to the understanding of the influence of the disordering on the optical properties of the amorphous state in phase-change alloys.

This study is aimed to reveal the compositional dependences in parameters that govern the optical properties of (GeTe)_x_(Sb_2_Te_3_)_1−x_ amorphous alloys in the wide spectral range from the phonons to the inter-band electronic transitions. By focusing on the absorption edge fluctuations that are linked to the variations of the bandgap *E*_*g*_, Tauc parameter *B*^*1/*2^, and the width of Urbach-Martienssen tails *E*_*U*_ in amorphous (GeTe)_x_(Sb_2_Te_3_)_1−x_ alloys, we expect to reveal the compositional trends in the influence of the structural and thermal disordering on the absorption processes in studied amorphous alloys.

## Results and Discussion

In the spectral range between phonons and below the absorption edge, real part of the dielectric function (DF) is governed by polarizability of bonded electrons and therefore can be described by the constant *ε*_*inf*_. In the case of covalently bonded compounds the dielectric constant satisfies the Clausius-Mosotti relation^3,13^ which expresses the dielectric constant *ε*_*inf*_ of material in terms of the atomic polarizabilities of the material’s constituent atoms.

**Figure 1:**
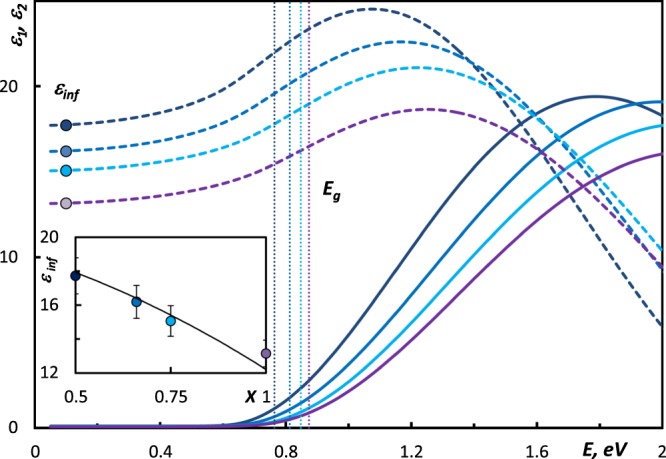
The dielectric function of amorphous (GeTe)_x_(Sb_2_Te_3_)_1−x_ samples: () Ge_1_Sb_2_Te_4_, () Ge_2_Sb_2_Te_5_, () Ge_3_Sb_2_Te_6_, () GeTe. Dashed lines correspond to the *ε*_1_, while solid lines correspond to the *ε*_2_. The dielectric constants *ε*_*inf*_ were determined as values of *ε*_1_ at 0.07 eV. The positions of the *E*_*g*_ are marked as vertical dashed lines and were determined by applying the α10k-criterion. Inset: The dielectric constants *ε*_*inf*_ of studied alloys. Solid line is calculated by applying the Clausius-Mossotti relation.

The DF of studied compounds shown in Fig. 1 was obtained from the fit of the measured spectra. The real part of the DF remains constant below the optical bandgap. The imaginary part of the DF of studied amorphous GSTs possesses nearly zero values at the energies below the bandgap, and then *ε*_2_ increases due to the electronic inter-band transitions with the range around the optical bandgap. Obtained dielectric constants *ε*_*inf*_ of studied samples follow the trend line estimated by the Clausius-Mossotti relation as shown in Fig. 1(I): *ε*_*inf*_ decreases while GeTe contents increases. For the calculation of the trend line we used the following values of the atomic polarizabilities^3^: *α*_Ge_ = 5.07 × 10^40^ Cm^2^ V^−1^, *α*_Sb_ = 7.82 × 10^40^ Cm^2^ V^−1^, and *α*_Te_ = 7.66 × 10^40^ Cm^2^ V^−1^. As in the range between phonons and the absorption edge, *ε*_*inf*_ is determined by the polarizabilities of the constituent atoms, the compositional evolution of the *ε*_*inf*_ is explained by the difference in atomic polarizabilities of Ge, Sb, and Ge atoms, whose concentration changes along the compositional line. In amorphous GSTs the majority atoms show covalent bonding and the coordinations around the majority atoms of Ge, Sb, and Te satisfy 8-N rules^3^.

Except for evolution of the *ε*_*inf*_, there are some more messages coming from Fig. 1. In the spectral range around 1.6 eV, the amplitude of the imaginary part of the dielectric function decreases as Ge content increases. The changes in the dispersion of *ε*_*2*_ in the range 0.6–1 eV indicate the shift of the optical absorption edge. These findings are in the agreement with theoretical and experimental data shown in^3,14^. The amorphous phase of GSTs is characterized by locally ordered motifs^10^, and long-range disorder with the volume expansion of 6% compared with the crystalline phases^14^. Altogether visually recognizable changes in the *ε*_*2*_ (Fig. 1) might indicate the evolution of the ordering that results in the changes of band-structure in the amorphous GSTs, which we now consider in more details.

The absorption coefficient *α* can be obtained from the equation α = 4πk/λ, where *k* is the extinction coefficient and *λ* is a wavelength^13^. From the perspective of fundamental material science the dispersion of the absorption coefficient can be divided into several spectral ranges. High-absorption region (*α* > 10^4^ cm^−1^) involves optical transitions between the valence and conduction band states, whereas the spectral range with 10^2^ < *α* < 10^4^ cm^−1^ is called the Urbach’s exponential tail region. Due to the lack of atomic long-range order that is caused by disordering the bond angles and lengths, amorphous materials do not have as sharp bandgap as crystalline materials have^13^. Therefore, the band structure of amorphous materials has to be complemented by localized states at the band edges and additional defect states inside the bandgap forming co-called the Urbach-Martienssen tails^15^. In the Urbach’s exponential tail region most of the optical transitions take place between localized tail states and extended band states. The region (*α* < 10^2^ cm^−1^) involves low-energy absorption which occurs due to the optical transitions between the localized states. The range around and slightly below the absorption edge in amorphous GSTs is of our interest. In the Urbach’s tail range, the absorption coefficient *α* shows an exponential dependence on photon energy *E*, and obeys the relation^15,16^:

1 $$\alpha ={\alpha }_{0}\,\exp \,(\frac{E-{E}_{0}}{{E}_{U}}),$$

where *α*_0_, is a constant, *E* is energy, *E*_0_ corresponds to the energy close to that of the bandgap at low temperature, and *E*_*U*_ is the Urbach energy, i.e. the width of the band tail of the localized states in the bandgap. Eq. 1 implies that logarithms of the absorption coefficient plotted as a function of the energy can be approximated by a straight line for the energies just below the fundamental absorption edge.

**Figure 2:**
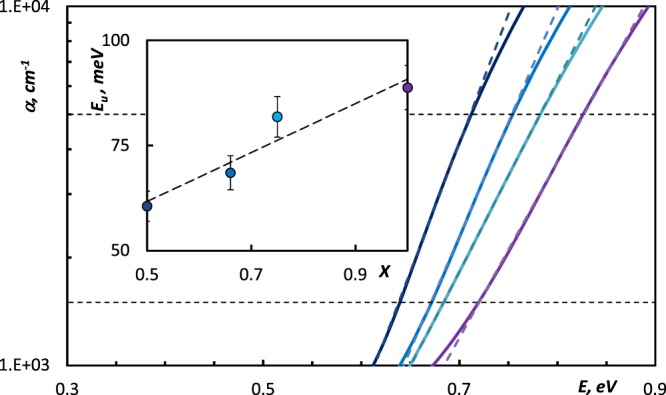
Dispersion of the absorption coefficients *α* in amorphous (GeTe)_x_(Sb_2_Te_3_)_1−x_ samples: () Ge_1_Sb_2_Te_4_, () Ge_2_Sb_2_Te_5_, () Ge_3_Sb_2_Te_6_, () GeTe. Straight dashed lines correspond to the linear approximation by using the Eq. 3. Inset: the compositional dependence in the Urbach energy *E*_*U*_ in (GeTe)_x_(Sb_2_Te_3_)_1−x_ samples. Straight trend line is shown for the visualization of the compositional dependence.

Figure 2 displays the absorption coefficients in amorphous GSTs which were derived from the data shown in Fig. 1. We show the linear approximation of the absorption coefficient as a function of the energy, for 1.5 × 10^3^ cm^−1^ < *α* < 5 × 10^3^ cm^−1^ in Fig. 2, where the value of *E*_*U*_ corresponds to the inverse slope of the approximating straight line. The value of Urbach energy *E*_*U*_ for amorphous Ge_2_Sb_2_Te_5_ obtained in the present study is consistent with corresponding value obtained in^17,18^. The evolution of the inverse slope of the approximating straight line reflects the systematic increase of the width of band tails of the localized states in the bandgap in amorphous materials along the Sb_2_Te_3_-GeTe line, as shown in the Inset of the Fig. 2.

To get the deeper insight into the nature of increase of the width of band tails in amorphous (GeTe)_x_(Sb_2_Te_3_)_1−x_, we now analyze the higher energies range above the absorption edge (*α* > 10^4^ cm^−1^), where the absorption is associated with inter-band electronic transitions, and the absorption coefficient obeys Tauc relation^19^:

2 $$\alpha E=B{(E-{E}_{G})}^{n},$$

where *B* is the Tauc parameter, *E* is photon energy, *E*_*g*_ is the optical bandgap and *n* is the number which relates to the mechanism of transition process. It was shown in^14,20,21^ that in amorphous (GeTe)_x_(Sb_2_Te_3_)_1−x_ alloys the photon energy is used for non-direct transition, that according to^22^ corresponds to *n* = 2. The Tauc parameter *B* represents the measure of the disorder in the system^23,24^. It includes the information on convolution of the valence band and conduction band states and on the matrix element of optical transitions, that reflects the ***k*** selection rule and the disorder-induced spatial correlation of optical transitions between the valence band and conduction band^25^. A decrease in *B*^*1/2*^ indicates an increase in the disorder^20,26^.

**Figure 3:**
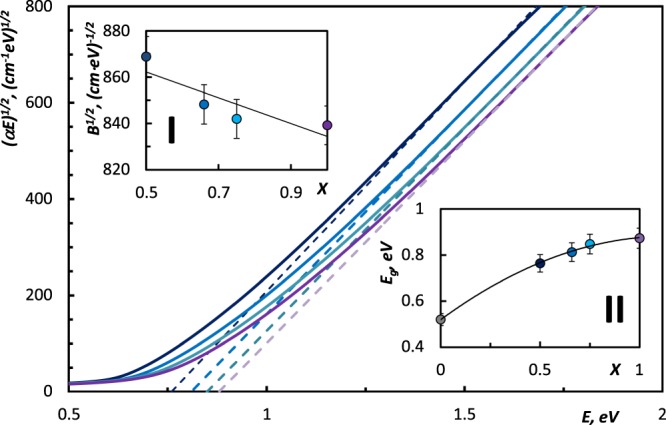
Plots of (*αE*)^1/2^ vs *E* for amorphous (GeTe)_x_(Sb_2_Te_3_)_1−x_ samples: () Ge_1_Sb_2_Te_4_, () Ge_2_Sb_2_Te_5_, () Ge_3_Sb_2_Te_6_, () GeTe. Dashed lines were calculated using the Eq. 1. Inset I: the compositional dependence in the Tauc parameter *B*^*1/2*^ in the (GeTe)_x_(Sb_2_Te_3_)_1−x_ samples. Straight trend line demonstrates the compositional dependence the Tauc parameter *B*^*1/2*^ (modification in short range order in GeTe-rich amorphous samples). Inset II: The optical bandgap *E*_*g*_ of the alloys. (*) the value of *E*_*g*_ of amorphous Sb_2_Te_3_ was taken from^14^. Trend line is shown for the visualization of the compositional dependence.

Figure 3 displays the plots of *αE* as a function of photon energy in the higher absorption range. The dashed lines were obtained from the Eq. 2. The interceptions of these lines with abscise correspond to the values of *E*_*g*_, which are in good agreement with values determined by *α*10 k criterion (the optical gap *E*_*g*_ that was also identified as the energy at which the absorption exceeds the value of 10^4^ cm^−1^, as shown in Fig. 1). The obtained results revealed the compositional dependence in the *E*_*g*_, as studied GST are mixtures of parent binary compounds, they inherit their properties in the corresponding proportion^27^. The values of the dielectric constant and the optical bandgap of Ge_1_Sb_2_Te_4_, Ge_2_Sb_2_Te_5_, and GeTe are in the quantitative consistency with data previously reported in^3^, where the samples have been prepared in the same manner using magnetron sputtering method. Trend in the evolution of the band-gap energies upon increasing GeTe content correlates with theoretical analysis of band-structure evolution in amorphous GSTs^14^. Obtained values of the optical band gap are consistent with results presented in other works. The previously reported values of optical bang-gap in amorphous Ge_1_Sb_2_Te_4_ vary from 0.69 eV to 0.76 eV^3,26^, in amorphous Ge_2_Sb_2_Te_5_ vary from 0.7 eV to 0.75 eV^18,26,28,29^, in amorphous GeTe they vary from 0.85 eV to 0.9 eV^12,30^. These minor discrepancies can be explained by the difference in the quality of samples obtained using different techniques. It is worth to mention, that the increase of the bandgap shown in the Fig. 3(II), is accompanied by the decrease of the dielectric constant *ε*_*inf*_ (Fig. 1, inset) in GeTe-rich compounds. This finding is reasonable, as both bandgap *E*_*g*_ and the dielectric constant *ε*_*inf*_ are linked via the empirical Moss rule in the following way^31^:

3 $${\varepsilon }_{\infty }^{2}{E}_{g}\approx const.$$

The slopes of the dashed lines in Fig. 3 are related to the corresponding values of *B*^*1/2*^ ^20^. We observe the decrement in *B*^*1/2*^ which reflects the increment in the randomness in atomic arrangement in the GeTe-rich amorphous materials from the studied part of the pseudo-binary Sb_2_Te_3_-GeTe line. This trend found to be in agreement with evolution of the widths of Urbach’s tails. It is worth to mention that obtained value of the Tauc constant for amorphous Ge_2_Sb_2_Te_5_ are higher than that reported in^20^ (for example, 831 vs. 516 at 300 K). This discrepancy can be explained by the different quality of the samples, as in^20^ samples were prepared using thermal evaporation method. However, both Urbach energy *E*_*u*_ and Tauc parameter *B*^*1/2*^ follow the compositional trends which are quite similar to those obtained in Sn-Sb-Se system with increase of concentration of Sn^32^.

**Figure 4:**
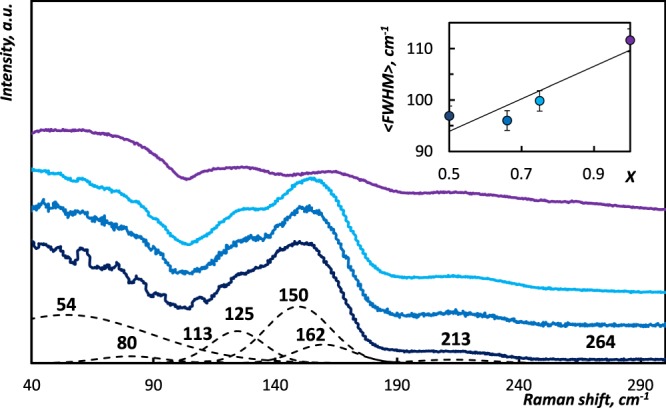
Raman spectra of amorphous (GeTe)_x_(Sb_2_Te_3_)_1−x_ samples: () Ge_1_Sb_2_Te_4_, () Ge_2_Sb_2_Te_5_, () Ge_3_Sb_2_Te_6_, () GeTe^10^. Dashed lines correspond to Gaussian decomposition of Raman spectrum of amorphous Ge_1_Sb_2_Te_4_ with positions of Gaussians marked^10^. Inset: The compositional increase of the average halfwidth <*FWHM*> in studied amorphous (GeTe)_x_(Sb_2_Te_3_)_1−x_ samples.

Optical and electrical properties of crystalline GSTs are sensitive to the presence of the disorder in the samples^5,33–37^. The presence of any kind of defects in material shortens the phonon lifetime. As the linewidth of a line in the spectrum is inversely proportional to the phonon lifetime, the presence of the disorder leads to the broadening of the linewidth. Raman spectra of amorphous (GeTe)_x_(Sb_2_Te_3_)_1−x_ samples, which are displayed in the Fig. 4, exhibit the similar pattern. In the previous work^10^ we analyzed the compositional dependencies in the intensities of bands in Raman spectra of amorphous (GeTe)_x_(Sb_2_Te_3_)_1−x_ samples. After the Gaussian decomposition procedure^38^, we focus on the evolution of the halfwidth *FWHM* in Raman spectra amorphous (GeTe)_x_(Sb_2_Te_3_)_1−x_ samples. The systematic increment in the average halfwidth <*FWHM*> (Fig. 4, Inset) results from the shortening of lifetime of Raman-active phonons caused by their increasing scattering due to the increasing imperfectness of the material.

Parameters that govern optical properties of the studied (GeTe)_x_(Sb_2_Te_3_)_1−x_ alloys at 300 K (Table 1) exhibit compositional evolutions. Increase in the width of localized states *E*_*U*_ that reflects in the widening of the optical bang-gap *E*_*g*_ is consistent with evolution of the Tauc parameter *B*^*1/2*^ and the average halfwidth <*FWHM*> of Raman bands. The compositional changes in *B*^*1/2*^ and <*FWHM*> evidence the increase of the disordering in the GeTe-rich amorphous (GeTe)_x_(Sb_2_Te_3_)_1−x_ alloys.

**Table 1:** Optical parameters of the studied (GeTe)_x_(Sb_2_Te_3_)_1−x_ alloys, 300 K.

	*ε*_*inf*_	*E*_*g*_, eV	*E*_*U*_, meV	*B*^*1*/*2*^, (cm·eV)^−1/2^	<*FWHM*>, cm^−1^
Ge_1_Sb_2_Te_4_	17.6 ± 0.05	0.76 ± 0.05	66 ± 3	869 ± 0.5	97 ± 2
Ge_2_Sb_2_Te_5_	16.3 ± 0.05	0.81 ± 0.05	73 ± 3	848 ± 0.5	96 ± 2
Ge_3_Sb_2_Te_6_	15.0 ± 0.05	0.86 ± 0.05	77 ± 3	842 ± 0.5	99 ± 2
GeTe	12.9 ± 0.05	0.89 ± 0.05	89 ± 3	839 ± 0.5	112 ± 2

To obtain further and deeper insight into the reasons that cause the changes in *E*_*U*_, we now try to determine the contributions of the structural (static) and thermal-induced (dynamic) disorder on the optical properties of amorphous (GeTe)_x_(Sb_2_Te_3_)_1−x_ samples by the analysis of the temperature-dependent data. An analysis of the contributions of the structural and thermal disorder into the variation of the optical absorption has been performed in^15,39^. The concept of equivalence of structural and compositional disorder has explained the relationship between the optical bandgap and Urbach energy^39^. An attempt to analyze the model of^39^ has been made^40^ to better understand the role of topological and thermal disorder in determining the optical bandgap in amorphous solids.

**Figure 5:**
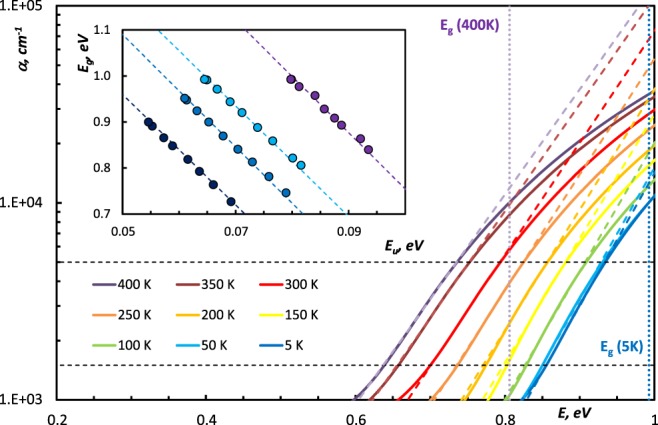
Temperature dependence of the absorption coefficient in amorphous Ge_3_Sb_2_Te_6_ with linear approximation according to Eq. 3. Horizontal dashed lines determine the range of the approximation. Vertical dashed lines indicate the position of the optical *E*_*g*_ at the extreme temperatures. Inset: Optical bandgap *E*_*g*_*(T)* as a function of *E*_*U*_*(T)* for studied (GeTe)_x_(Sb_2_Te_3_)_1−x_ alloys: () Ge_1_Sb_2_Te_4_, () Ge_2_Sb_2_Te_5_, () Ge_3_Sb_2_Te_6_, () GeTe. This dependence demonstrates correlation between *E*_*g*_*(T)* and *E*_*U*_*(T)*.

In Fig. 5, one can clearly distinguish the blue-shift of the absorption edge in amorphous Ge_3_Sb_2_Te_6_ at low temperatures, that reflects the significant widening of the bandgap in the studied temperature range (17–19%) shown in Inset in Fig. 5. Variation of the energy bandgap with temperature arises from the shift in the relative position of the conduction and valence bands due to dilatation of the lattice and due to temperature-dependent electron-lattice interaction^41–44^. Due to thermal dilatation of lattice, an increased interatomic spacing decreases the potential seen by electrons in the material, which reduces the width of the energy bandgap. To analyze the temperature dependence of the optical bandgap we used the following expression^39^:

4 $${E}_{g}(T)={E}_{g}(0)-\beta {(exp(\theta /T)-1)}^{-1},$$

where *T* is the sample temperature, *E*_*g*_*(*0*)* is the bandgap energy at *T* = 0 K, *β* is the material constant linked to the second-order deformation potential^15,39^, and *Θ* is characteristic Einstein temperature.

The blue-shift of the absorption edge and the corresponding dependence *E*_*g*_(*T*) is not the only feature notable in Fig. 5. We approximated the temperature evolution of the absorption coefficient by straight lines according to Eq. 3 for 1.5 × 10^3^ cm^−1^ < *α* < 5 × 10^3^ cm^−1^. These lines for various temperatures are not parallel and they converge at a point (*E*_0_*,α*_0_), called the “converging point”^45^. As the inverse slope of the approximating straight line corresponds to the Urbach energy *E*_*U*_, this fact evidences the temperature dependence *E*_*U*_*(T)*. The similarity between the temperature dependences of *E*_*g*_ and *E*_*U*_ is illustrated in the Inset in Fig. 5 where *E*_*g*_*(T)* is plotted against *E*_*U*_*(T)*, with temperature as a parameter. The linear relationship between *E*_*g*_ and *E*_*U*_ for all studied compounds (Table 2) confirms that *E*_*g*_*(T)* and *E*_*U*_*(T)* have the same functional form, as shown^15^.

**Table 2:** Temperature dependences of the Urbach energy and the optical bandgap in amorphous (GeTe)_x_(Sb_2_Te_3_)_1−x_ alloys. (The errors in determining the values of the Urbach energy and the optical bandgap are ±1 meV and ±0.05 eV, correspondingly).

*T, K*	Ge_1_Sb_2_Te_4_		Ge_2_Sb_2_Te_5_		Ge_3_Sb_2_Te_6_		GeTe	
	*E*_*U*_, meV	*E*_*g*_, eV	*E*_*U*_, meV	*E*_*g*_, eV	*E*_*U*_, meV	*E*_*g*_, eV	*E*_*U*_, meV	*E*_*g*_, eV
5	55	0.90	61	0.95	65	0.99	80	0.99
50	55	0.89	61	0.95	65	0.99	80	0.99
100	57	0.87	63	0.92	67	0.97	81	0.98
150	59	0.85	65	0.90	69	0.94	84	0.96
200	62	0.82	68	0.87	71	0.92	86	0.93
250	64	0.79	70	0.84	74	0.89	88	0.91
300	66	0.76	73	0.81	77	0.86	89	0.89
350	69	0.73	76	0.78	80	0.82	92	0.86
400	71	0.70	79	0.75	82	0.81	94	0.84
*ΔE*_*U*_ */E*_*U(5K)*_	0.309		0.294		0.264		0.172	
*ΔE*_*g*_ */E*_*g(5K)*_	−0.223		−0.216		−0.188		−0.154	

In order to estimate the temperature dependence of the Urbach tails, we applied the following expression^15^:

5 $${E}_{U}(T)={E}_{U(st.)}+{E}_{U(dyn.)}=\frac{{k}_{B}\theta }{{\sigma }_{0}}(\frac{1+X}{2}+\frac{1}{exp(\theta /T)-1}),$$

where *E*_*U(st.)*_ is contribution of structural disorder; *E*_*U(dyn.)*_ is the contribution of thermally-induced disorder*, k*_*B*_ is Boltzmann constant, *Θ* is characteristic Einstein temperature, *X* is a measure of structural disorder normalized to the zero-point uncertainty in the atomic positions^15^, and *σ*_*0*_ is Urbach edge parameter of order unity, which is inversely proportional to the strength of the coupling between electrons and phonons^45^. For the purpose of this study, the characteristic Einstein temperature is a good approximation to the temperature corresponding to the average phonon energy, as reported^20^.

**Figure 6:**
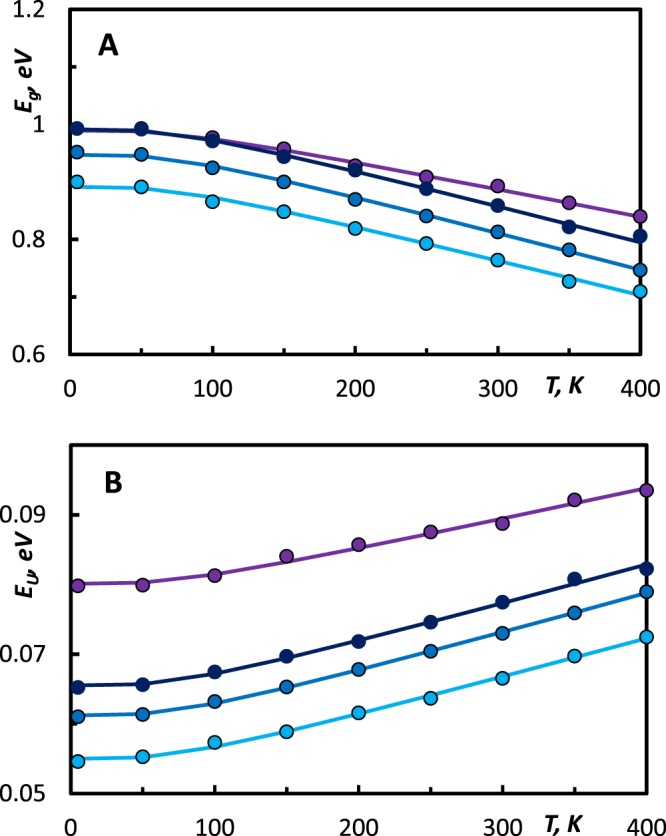
(**A**) Optical bandgap *E*_*g*_ as a function the temperature. Dots correspond to the values of *E*_*g*_ obtained by applying the α10k-criterion to the dispersion of the absorption coefficient. Solid lines were calculated using Eq. 4. (**B**) Urbach energy *E*_*U*_ as a function the temperature. Dots correspond to the values of *E*_*U*_ obtained from Eq. 3, solid lines were calculated using Eq. 5. () Ge_1_Sb_2_Te_4_, () Ge_2_Sb_2_Te_5_, () Ge_3_Sb_2_Te_6_, () GeTe.

As shown in Fig. 6(A), *E*_*g*_(*T*) dependence exhibits the parabolic shape at low temperatures (below the Debye temperature) and linear shape at higher temperatures. The Debye temperatures of these alloys are in the range of 110–125 K^46,47^. Before applying fitting procedures to the experimental dependences *E*_*g*_(*T*) and *E*_*U*_(*T*) we evaluated them by calculating *ΔE*_*U*_/*E*_*U(5K)*_ and *ΔE*_*g*_/*E*_*g(5K)*_. These values shown in the Table 2 demonstrate following compositional trends: GeTe-rich amorphous (GeTe)_x_(Sb_2_Te_3_)_1−x_ alloys exhibit less pronounced temperature dependences of the optical bandgap and Urbach energy, the latter may serve as an additional evidence of the increase of the disordering in these compounds. We found and show in the Fig. 6 that the fitting *E*_*g*_*(T)* and *E*_*U*_*(T)* by Eqs 4 and 5 using set of parameters shown in the Table 3 is quite satisfying for tracing the experimental data.

**Table 3:** Fit parameters of *E*_*U*_ *(T)* and *E*_*g*_*(T)* in (GeTe)_x_(Sb_2_Te_3_)_1−x_ alloys.

	Ge_1_Sb_2_Te_4_	Ge_2_Sb_2_Te_5_	Ge_3_Sb_2_Te_6_	GeTe
*E*_0_ *, eV*	0.89	0.95	0.99	0.99
*Θ, K*	209.4	209.4	209.4	209.0
*β, eV*	0.13	0.14	0.13	0.10
*σ*_0_	1.52	1.49	1.51	1.91
*X*	8.25	9.13	9.88	16.01

The contribution of thermally-induced disorder *E*_*U(dyn.)*_ is controlled by thermal lattice vibrations, and, hence it increases upon increasing temperature. Ternary (GeTe)_x_(Sb_2_Te_3_)_1−x_ alloys in the amorphous state possess equal average phonon energies, and they do not exhibit any significant compositional trends in the degree of phonon anharmonicity as shown by results of temperature-dependent IR and Raman spectroscopy^10,11^, and therefore we can conclude that the contribution of the thermally-induced disorder *E*_*U(dyn.)*_ to *E*_*U*_*(T)* should be alsmost equal for all studied amorphous (GeTe)_x_(Sb_2_Te_3_)_1−x_ alloys without any compositional dependences.

The contribution of structural disorder *E*_*U(st.)*_ arises from the lack of the long range order in amorphous (GeTe)_x_(Sb_2_Te_3_)_1−x_ alloys. Therefore, the variation of the disorder in studied samples should reflect in evolution of *E*_*U(st.)*_. The fit of *E*_*U*_*(T)* with Eq. 5 yields the clear compositional increment in the fit parameter *X*, as shown in the Table 3. While moving along the pseudo binary line towards GeTe, the concentration of the vacancies reduces, latter leads to the broader distributions of bond lengths and bond angles^48^ in corresponding amorphous (GeTe)_x_(Sb_2_Te_3_)_1−x_ alloys.

## Conclusions

In summary, we observed systematic trends in the optical properties of amorphous (GeTe)_x_(Sb_2_Te_3_)_1−x_ alloys in the IR. The dielectric constant *ε*_*inf*_ obeys the Clausius-Mossotti relation and can be determined by the polarizabilities of the material’s constituent atoms. The evolution of the dielectric function around the fundamental absorption edge reveals the systematic changes in the optical bandgap width *E*_*g*_ and in the width of the localized electronic states *E*_*U*_. The compositional increment in *E*_*U*_ can be explained by increasing of the disorder in GeTe-rich amorphous (GeTe)_x_(Sb_2_Te_3_)_1−x_ alloys. This conclusion is supported by corresponding trends in the Tauc parameter *B*^1*/*2^ and in the average halfwidth <*FWHM*> of Raman bands. The low-temperature data enabled us to analyze the contributions of the structural and thermal-induced disorder into the temperature dependence of *E*_*U*_ and *E*_*g*_. While no compositional trends in the contribution of thermal-induced disorder can be traced, there is a systematic increase of the structural disorder in GeTe-rich amorphous (GeTe)_x_(Sb_2_Te_3_)_1−x_ alloys. This might be due to the fact that reducing the concentration of vacancies in GeTe-rich GSTs leads to the broader distributions of bond lengths and bond angles that worsen long-range ordering in studied amorphous (GeTe)_x_(Sb_2_Te_3_)_1−x_ alloys. Obtained results may enable one to optimize the tailored optical properties of amorphous (GeTe)_x_(Sb_2_Te_3_)_1−x_ alloys. The latter is quite important for their technological applications which require the long-term stability and the absence of the drift effects.

## Methods

### Samples preparation

In this study we have chosen several phase-change alloys along the (GeTe)_x_(Sb_2_Te_3_)_1−x_ compositional line: Ge_1_Sb_2_Te_4_ (*x* = 0.5), Ge_2_Sb_2_Te_5_ (*x* = 0.66)_,_ Ge_3_Sb_2_Te_6_ (*x* = 0.75), and GeTe (*x* = 1). To prepare the samples for optical measurements, the 150 nm Al layer was deposited onto a glass substrate. The phase-change films (1,000 nm) were d.c. sputtering-deposited with LS 320 von Ardenne system (background pressure 4 × 10^−7^ mbar, 20 s.c.c.m. Ar flow, deposition rates 0.1–0.2 nms^−1^, operating in the constant power mode by using 20–25 W) with stoichiometric targets of 99.99% purity. In each sputter session 4 samples of a certain compound have been prepared: 3 samples were used for the optical measurements to provide the repeatability of the data, one reference sample w used to determine the thickness of the phase-change layer by using a profilometer.

### Optical measurements

Reflectance spectra were measured within the energy range from 0.05 up to 1 eV with a resolution of 2 meV, using Bruker IFS 66 v/s spectrometer equipped with the continuous flow Cryovac cryostat, in the sample temperature range from 5 up to 400 K. As a reference sample, a glass substrate coated with the 150 nm Au layer was used, it acted as an almost ideal mirror in the studied energy range. Reflectance spectra of the reference sample and the studied one were measured subsequently to exclude drift effects. For normalization, the final spectrum was obtained by dividing the measured spectrum by the reference one. The angle of incidence of the incoming beam was kept constant at 10° with respect to the surface normal. To extend the studied spectral range we measured ellipsometry spectra in the range from 0.7 to 2 eV using a Woolam M-2000 UI with angles of incidence of 60 and 69 at room temperature. The ellipsometer was equipped with a InGaAs diode array, as well as silicon charge-coupled device camera that served as detectors in the NIR and the VIS/UV ranges correspondingly. The resolution was of about 7 meV. The light sources were deuterium and halogen lamps.

The relative measurement error for the optical measurements was 0.2% in the used wavelength range.

### Modeling the spectra

Optical spectra were analyzed using the SCOUT and CoRa software. The model of the dielectric function *ε*(*ν*) for the studied samples was composed of the *ε*_*inf*_, a real constant that describes the polarizability of bonded electrons, which plays a major role in the energy range above the phonons. The inter-band electron transitions were described by the OJL model^49^ which includes tail states exponentially decaying into the band gap. The dielectric constant *ε*_*inf*_ was determined at 0.1 eV.
